# A Smart, Data-Driven Approach to Qualify Additively Manufactured Steel Samples for Print-Parameter-Based Imperfections

**DOI:** 10.3390/ma17112513

**Published:** 2024-05-23

**Authors:** Suresh Alaparthi, Sharath P. Subadra, Shahram Sheikhi

**Affiliations:** 1 Institute of Materials Science and Joining Technology, University of Applied Science Hamburg, Berliner Tor 13, D-20099 Hamburg, Germany; 2 Forschungs-und Transferzentrum 3i, University of Applied Science Hamburg, Berliner Tor 13, D-20099 Hamburg, Germany

**Keywords:** additive manufacturing, gas metal arc welding, microstructure, porosity, defects, quality, non-destructive testing, acoustics, data acquisition, sorting algorithm

## Abstract

With additive manufacturing (AM) processes such as Wire Arc Additive Manufacturing (WAAM), components with complex shapes or with functional properties can be produced, with advantages in the areas of resource conservation, lightweight construction, and load-optimized production. However, proving component quality is a challenge because it is not possible to produce 100% defect-free components. In addition to this, statistically determined fluctuations in the wire quality, gas flow, and their interaction with process parameters result in a quality of the components that is not 100% reproducible. Complex testing procedures are therefore required to demonstrate the quality of the components, which are not cost-effective and lead to less efficiency. As part of the project “3DPrintFEM”, a sound emission analysis is used to evaluate the quality of AM components. Within the scope of the project, an approach was being developed to determine the quality of an AM part dependent not necessarily on its geometry. Samples were produced from WAAM, which were later cut and milled to precision. To determine the frequencies, the samples were put through a resonant frequency test (RFM). The unwanted modes were then removed from the spectrum produced by the experiments by comparing it with FEM simulations. Later, defects were introduced in experimental samples in compliance with the ISO 5817 guidelines. In order to create a database of frequencies related to the degree of the sample defect, they were subjected to RFM. The database was further augmented through frequencies from simulations performed on samples with similar geometries, and, hence, a training set was generated for an algorithm. A machine-learning algorithm based on regression modelling was trained based on the database to sort samples according to the degree of flaws in them. The algorithm’s detectability was evaluated using samples that had a known level of flaws which forms the test dataset. Based on the outcome, the algorithm will be integrated into an equipment developed in-house to monitor the quality of samples produced, thereby having an in-house quality assessment routine. The equipment shall be less expensive than conventional acoustic equipment, thus helping the industry cut costs when validating the quality of their components.

## 1. Introduction

Additive manufacturing (AM) techniques have evolved from rapid prototyping techniques to a widely used manufacturing process with unique benefits over conventional processes [1,2]. According to the ISO/ASTM 52900 [3] norm, additive manufacturing (AM) is the general term for techniques in creating components by adding material repeatedly (layer-by-layer) while adhering to a computer-aided design model. Layers of metallic powder are melted using a concentrated heat source in an iterative process to create a component [4].

Due to its high material deposition and construction speeds, Wire Arc Additive Manufacturing (WAAM) is one of the various AM techniques that is accessible and is particularly helpful for making large parts [5]. To explain the procedure in more detail, metallic wire (feedstock) is continuously melted using an electric arc to create structures. There are various welding procedures through which WAAM can be realized; the most commonly utilized ones are Gas Metal Arc Welding (GMAW) and Gas Tungsten Arc Welding (GTAW). The former is better than the latter since the wire serves as a consumable electrode, reducing the amount of equipment needed and the manufacturing process. According to Wu et al. (2018) [6], WAAM is a dependable technology for a number of sectors because it has been used with numerous metals, including steel, titanium, nickel, and superalloys. Ya and Hamilton (2018) [7] examined the maritime industry’s propeller manufacturing process, examining several propeller designs and processing circumstances.

Even though the finished product produced using WAAM-manufacturing techniques has mechanical qualities that are similar to those parts produced using conventional techniques, there are certain flaws that need to be fixed. It is necessary that we prevent porosity, excessive residual stress levels, cracking, and other issues in order to utilize the part in harsh conditions. Poor programming techniques, unstable weld pool dynamics, thermal deformation with heat buildup, and other machine failures are the main causes of these errors [6]. The ultimate product quality is determined by these elements. Respecting quality standards is vital and difficult when dealing with complicated geometries, particularly when those geometries include elements that are inaccessible and cannot be verified by traditional metrology methods. Deep faults have a significant impact on quality, and, when combined with the intricate shape, they make the non-destructive method an excellent way to raise the calibre of products manufactured through additive manufacturing [8].

Non-destructive testing (NDT) has been widely used in industry and academia to determine product performance and quality. Various techniques have been developed over time [1] to comprehend the existence of flaws in materials. A few of the techniques are thermography, acoustic emission, ultrasonic, electromagnetic, radiography, impulse stimulation, and dye-penetrant methods. Even though X-ray computed tomography (XCT) is the gold standard for evaluating quality, this process is costly and time-consuming [8,9]. The resonant response of materials and the components formed of those materials is the basis of the resonant frequency method (RFM), which has been in use for a number of years. In this case, the test object is mechanically excited (either by impact or sweep excitation) in order to excite the resonance frequencies, following which the response is recorded (either by a contact sensor or microphone). After the signals are obtained, a variety of filtering and transformation techniques are applied to extract different sound properties, including damping characteristics, amplitudes, and eigenfrequencies. By using an appropriate algorithm, which may separate good and bad samples based on sound characteristics, in addition to carrying out the filtering and transformation, operations can also be realized [10].

Cross-referencing with reference part frequencies was a vital methodology used to explore the state of the art on RFM methodologies to classify defective and non-defective WAAM components; a divergence from these (reference frequencies) is interpreted as a flaw in the component. Obatan, AF et al. [11] used data analytics techniques to efficiently analyze resonant ultrasound signals for defect identification and classification, then used resonant frequency algorithms to locate the missing struts in complicated AM samples. According to the tests carried out for this research, it was found that the reference sample used for defect identification is presumed to be faultless based on the data that are currently accessible [11]. We plan to investigate this methodology further in this research by using a 3D-printed sample as a reference. Since the sample will be precisely machined, geometry will not have an impact on the frequency deviations. The intentional introduction of faults into the sample would result in frequency shifts; the concentration of defects would be determined by ISO 5817 (Welding—Fusion-welded joints in steel, nickel, titanium, and related alloys—Quality levels for imperfections) [12]. It should be emphasized that traditional welding standards will be utilized for this work as there are currently no ISO/ASTM standards available to quantify flaws in WAAM samples.

Certain businesses have created algorithms using statistical data to classify faulty components using outlier detection—a method that relies on references-to-samples that are healthier. The majority of these systems rely on training procedures relevant to particular applications that are based on empirical data. This means that, in order to cover all potential abnormalities, a training dataset must be created beforehand by measurements of representative sections. Due to the necessity to create numerous reference samples and account for all potential anomalies, this is a costly and intricate procedure. The two main drawbacks of this approach are that the algorithm has to be retrained using the new dataset, and the dataset needs to be updated to account for any new errors that may be discovered over time. Thus, it can be claimed that creating a database through experimentation alone in order to train an algorithm that would eventually be able to distinguish flawed 3D-printed components might be costly and require a lot of human resources. Thus, in order to create the largest dataset feasible in terms of frequency change, we combine an experimental and a simulation-based technique in this study. We next use the dataset to train a regression modelling algorithm. Subsequently, the algorithm might be incorporated into a tool designed especially for 3D-printed sample quality checking [10].

Though there exists a lot of possibilities in the realm of acoustics to classify healthier samples from unhealthier ones, the very cost of such equipment and their purpose limit them. Through this research funded by Bundesministerium für Wirtschaft und Klimaschutz (BMWK), we intend to develop a piece of equipment with the requisite sorting algorithm embedded into the system. Such a piece of equipment is cost-effective, and can be used for different sample sets based on the user’s requirement (print-parameter-based defects can be ascertained). Though geometry can be a limiting factor for such an equipment, in the case of AM sub-types such as WAAM, print parameters are a crucial aspect. Therefore, before the geometric aspect is explored, the print-parameter-induced defects such as porosities need to be studied and appropriate conventions established. Developing a piece of equipment is the goal of this BMWK-funded project, whereas, through this article, the authors intend to establish a procedure that can be useful in helping qualify WAAM samples. The procedure is based on ISO-5817, which shall be the basis for the development of a machine-learning algorithm based on regression modelling to sort WAAM samples based on the level of porosity in them. To the knowledge of the authors, very little has been carried out in this regard to qualify samples, and, hence, this article is an attempt to rectify it.

The paper is divided into four sections including the introduction. Section 2 outlines the materials and methodology adopted, where the sub-sections cover various aspects like sample preparation, density measurements/metallography, FEM methodology and resonant frequency method/equipment, data acquisition, and the development of the algorithm. With regard to the algorithm, the methodology of classification is explored along with the development of the training dataset. Subsequently, details on the training set is explained. Section 3 discusses the results, where the density and metallography are explained first; later, the results from FEM are elaborated. Further ahead in the section, the algorithm is picturized, and the functionality delved into. Lastly, in the section, the algorithm’s ability to classify samples with defects is shown and its feasibility investigated through the testing dataset. The paper concludes with Section 4.

## 2. Methods

### 2.1. Sample Preparation from WAAM

A commercial steel (S355) block with a size of 250 × 80 × 100 mm^3^ was selected as the substrate plate. The filler wire 316LSi with a diameter of 1.0 mm was used to deposit the components in the WAAM process. The 316LSi wire exhibits elevated silicon content compared to 316L, aiming to enhance the fluidity of the molten material and, consequently, improve weldability. The chemical compositions are given in Table 1 [13].

**Figure 1:**
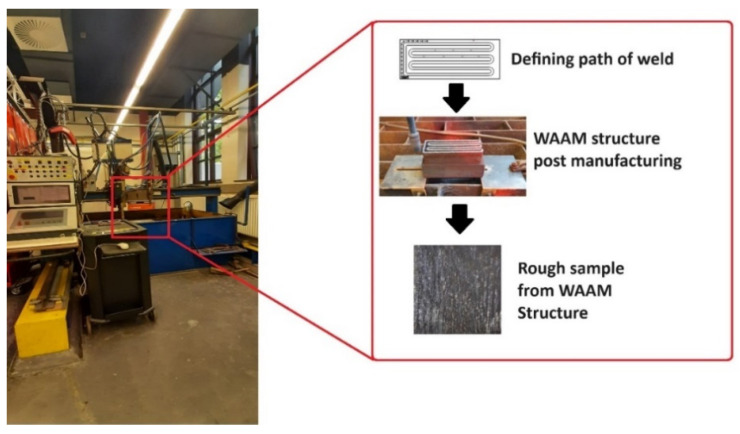
Step 1: WAAM Structure Production: Defining the path for print, obtaining the WAAM structure, and, lastly, cutting the rough sample for milling.

The WAAM process was performed on a water-cooled iron frame (WAAM can be seen in Figure 1). The manufacturing of the parts maintained consistent process parameters throughout the entire study. These parameters included a current of 112 A, a travel speed (TS) of 0.6 m/min, a wire feed speed (WFS) ranging from 5.4 to 6.8 m/min, and a cooling time of 15 min after each layer. The shield gas employed consisted of 2% CO_2_ and 98% argon. The height of each layer was 1.2 mm and each layer consists of 6 straight lines and torch moving in a meandering-like path. A total of 51 layers were printed and Figure 1 illustrates the WAAM-manufacturing process pictographically as Step 1 of the final sample production.

**Figure 2:**
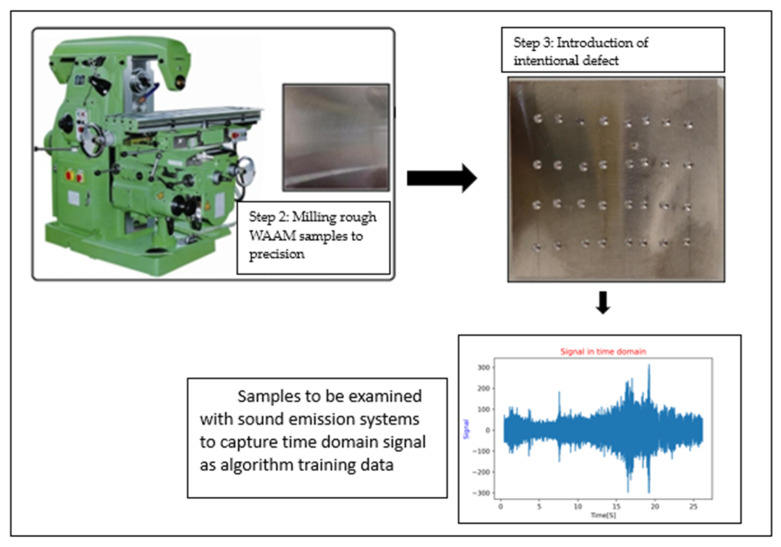
Stages of sample production and testing.

Following the printing process, the layers were detached from the substrate using saw-cutting equipment. Subsequently, a total of 6 lines were cut, resulting in 18 samples, with 3 taken from each line. These samples were then milled to achieve dimensions of 51.8 × 50.0 × 6 mm^3^. Further refinement was carried out by milling to surface-smooth the samples, ultimately reaching final dimensions of 51.8 × 50.0 × 3.95 mm^3^. The defects in the samples were intentionally introduced through a manual process involving the use of an automated drilling machine. The drilling operation utilized a drill with a diameter of 1 mm, and a consistent depth of 1 mm was maintained for each hole. Subsequent to drilling, frequency measurements were recorded to assess the impact of the introduced defects on the samples. It must be stated that the defect in the form of holes is quantified as porosity in this paper. This was adopted since precisely picturizing and quantifying defect (porosity) would require a CT-Scan. Due to an unanticipated delay in delivery of the equipment, it was decided to adopt this methodology of introducing holes and quantifying the defect in terms of porosity. Figure 2 shows these processes in stages, where, initially, the WAAM samples are milled to precision, followed by introduction of defects which would then be tested for its resonant frequencies. The data are later used to train the algorithm.

### 2.2. Density Measurements

Density measurements were conducted employing both metallographic and Archimedes’ Principle methods to comprehensively assess the density characteristics of the samples. The metallographic method involved evaluating the sample density for a specific plane in the micro-section, providing insights into localized variations. In contrast, Archimedes’ Principle offered an integrated density assessment for the entire sample volume. The methodology adhered to ASTM B962 [14] standards, where samples were initially weighed in air, and subsequently suspended in distilled water to determine their density relative to the theoretical density of 316L stainless steel is 7.99 g/cm^3^ [15], accounting for the density of water at the specified temperature. Additionally, the presence of air bubbles on the sample surface was meticulously examined to ensure measurement accuracy. The mass of the printed parts in both air and water was measured thrice using an electronic balance, and the average mass was computed. Utilizing Equation (1):

(1) $$Density=\frac{Ma}{\left(Ma-Mw\right)}\times dw$$

where *Ma* represents the mass in air, *Mw* is the mass in water, and *dw* is the density of water, the density of each sample was determined.

In the metallographic analysis, the samples underwent a series of preparation steps. Initially, they were embedded in Bakelite, and then subjected to grinding and polishing processes to achieve a suitable surface for examination. Subsequently, the inherent grain structure of the samples was meticulously examined using the Keyence VHX-6000 optical microscope (Kansas City, MO, USA). This advanced microscope, equipped with a zoom capability ranging from 50 to 1000 µm, allowed for a comprehensive and detailed analysis of the samples’ grain morphology, specifically after the etching process had been applied.

### 2.3. FEM Method for Database Enhancement

The resonant frequencies were ascertained through FEM simulations using a CAE software (Ansys workbench 2020 R2 version). Quadratic 3D elements were used for generation of mesh in the simulated parts, whereas, for the mesh size, two distinct element sizes were used. In the case of parts without any defects, a larger mesh size of 1 mm was adopted, while, for defected parts, a mesh size of 0.3 mm was used (tetrahedral). The latter size was adopted to enable accurate representation of local changes in geometry. The reduction in mesh size could capture the effects of defects on frequencies more precisely [16]. Another important aspect to be stated here is that the arrangement of the holes was carried out as per the location of the holes on the experimental samples. This means the location of the holes on the samples were marked, and the same location was used on the FEM samples, so as to remain consistent. Material properties highlighted in Table 2 were adopted.

Initially, the samples were designed on Autodesk Fusion 360 (San Francisco, CA, USA) and imported as STEP files into the geometry section of the modal analysis module of the CAE software; the sample dimensions were kept constant at 51.8 × 50.0 × 3.9 mm^3^. The material parameters elaborated in Table 2 were input in the engineering section of the module. The integration of porosity as a defect into CAD design was achieved as per measurements carried out using an optical microscope. The volume fraction of the defect matched the percentage adopted in the paper. The FEM study serves two purposes in this study: first, it helps in having a credible dataset of frequencies for the algorithm to be trained; and, second, it assists in identifying frequencies associated with unwanted modes which are not necessary in this study. A parametric study would be carried out with varying E-modulus values and a fixed geometry. Thus, it enables us to identify the correct frequencies which are in proximity to those seen while conducting experiments [17].

### 2.4. Resonant Frequency Measurement and Developing the Alogrithm

#### 2.4.1. Brief Overview of the Methodology

**Figure 3:**
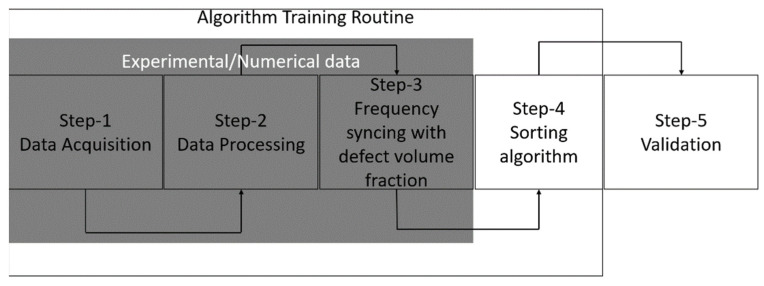
An overview of the methodology adopted to collect data and to subsequently train the algorithm.

Figure 3 presents the process and describes how a dataset (obtained from experiments and simulations) can be used to train the algorithm to sort/classify defective samples. This allows for the elaboration of the following:

Step 1: Gathering data from both experimental and FEM means is part of the data-collecting process. According to ISO 5817, the maximum allowable limit (category D) of defects within a weld structure shall not exceed 2.5%. When the maximum level of quality (category B of ISO 5817) is to be attained, the defects shall not exceed 1% of the overall area. This article implements a comparable defect distribution and records the corresponding frequencies. The datasets initially were time-domain signals that were collected using RFM apparatus. To acquire a similar set of data, samples with and without flaws were simulated in parallel using the FEM software (Ansys workbench 2020 R2 version). In this case, it would also be explored how the simulation and experimental data offset each other.

Step 2: Data processing entails employing Fast Fourier transforms to process the data, which are essentially time-domain signals, into frequency-domain signals. The processed database (training set) is formed by selecting and storing the frequencies in a file as a point of reference. The algorithm’s training dataset would come from this database.

Step 3: A volume fraction of defect is assigned to the frequencies created in the previous step after they have been sorted. Section 2.4.3 and Section 2.4.4 would further elaborate on this.

Step 4: Using the database created in the previous step as a basis, the algorithm is put into practice. All of the references to defect percentage that are detailed in ISO 5817 are contained in this database.

Step 5: The last step involves validation of the developed algorithm with random WAAM samples with intentional defects of unknown percentage. The next few sections shall further elaborate the steps in terms of the methodologies adopted and the final classification to be carried out.

#### 2.4.2. Resonant Frequency Measurement Equipment

ISO EN 843 [18] provides details on the tools that are available for determining resonance frequencies. This standard provides information for determining the elastic moduli, especially the E-modulus, shear modulus, and Poisson’s ratio from the fundamental natural frequencies of the materials considered. The methodology behind this approach is straightforward: samples with similar materials and geometry will have similar frequency spectra, while a slight change in its material composition/geometry causes a change in the spectra. This can be observed by checking the peak frequency shifts, and a statistical study on several samples can help this shift be validated correctly. Thus, this method is a useful tool to monitor structural changes within any component. The validity of this method is dependent on the number of samples: the higher the number, the better the results [2,3,4,19].

**Figure 4:**
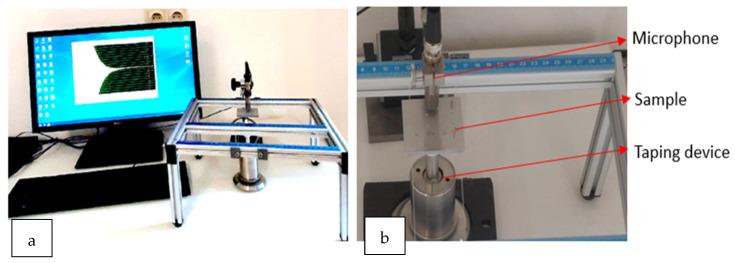
Experimental setup for data acquisition: (**a**) computer along with data acquisition, and (**b**) taping system along with microphone and suspended sample.

In the experimental procedure, the setup is as shown in the figure below. Figure 4a shows a computer along with the data acquisition systems, and, in Figure 4b, one can see a custom-made table with strings on which the sample can be suspended along with a taping device below the sample and a microphone above it. The test piece is suspended on threads along the edges slightly away from the nodes of vibration, and the microphone is placed along the central axis also termed the anti-node [18]. The detected sound is transduced to electric signal, which is amplified, and a separate algorithm was developed to perform Fast Fourier Transform to estimate the dominant frequency from generated sound signal, and, hence, a power spectrum is generated. The algorithm can detect the required resonant frequencies from the spectrum which it does based on a set frequency range which it calculates by itself based on the sample dimensions and mechanical properties referenced from literature (from a database with all Young’s modulus values).

#### 2.4.3. Experimental Data (Signal) Acquisition and Processing

**Figure 5:**
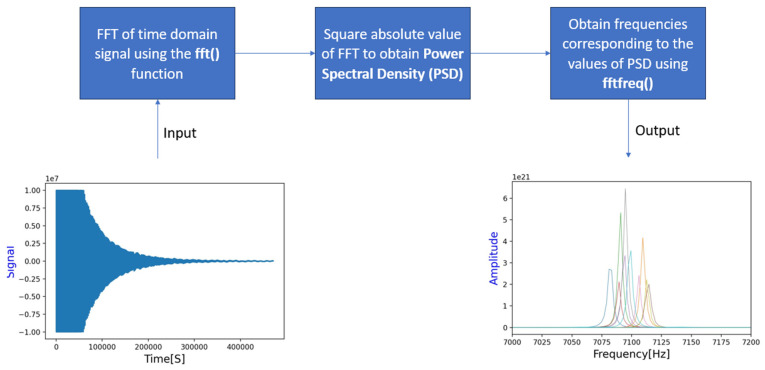
Step-by-step process to implement a time-to-frequency conversion.

A typical time-domain signal is shown in Figure 5 (bottom left), which shows the variation of the signal with respect to time. The signal can be converted into a frequency domain by performing a Fast Fourier transform (seen in Figure 5, bottom right), and the plot generated is called a spectrum and shows the frequency content of the time-domain signal. This implies how much of the signal lies in each frequency band over a range of frequencies. For the sake of analysis, the frequency domain was preferred over time domain in this paper because the latter tends to change rapidly over a short period of time, and, hence, interpreting such changes can be tiresome. The sampling rate is an important factor while performing time-to-frequency domain conversions via FFTs. The sampling rate in our case was 200 kHz, and the spectrum obtained shall have a certain resolution, and this is estimated using the following equation:

(2) $$∆f=\frac{f_{s}}{N}$$

where $f_{s}$ is the sample vibration frequency and *N* the number of points taken out of the signal for calculation of the spectrum. When *N* becomes smaller, the resolution becomes bigger. The FFT calculation takes time, and this is minimal when *N* is chosen as a power of 2. When the type of sample stays the same (same signal length) and the sampling rate is kept constant, the spectra will have the same resolution [20]. The process within the algorithm to achieve time-to-frequency domain involves few important steps. The first few steps involve setting up the .csv files, loading them into a data-frame and generating the plots. The second few steps involve setting up the time-step to determine the number of samples per second for FFT and, lastly, performing the FFT and generating a spectrum with distinct peaks which establishes the frequency content within the time-domain signal. This is shown pictorially below, where the time signal is read first. Thereafter, a set of operations within the algorithm converts the time to frequency domain. There are several packages available to perform FFTs within Python: a few examples are Numpy, Scipy [21], etc. A clearly defined sampling rate should perform the conversion correctly with a clear spectrum.

#### 2.4.4. Overview on the Development of the Algorithm

To further elaborate on the methodology to classify samples based on their defects, ISO 5817:2014 was referred to and used as the basis. It should be stated here that this standard is a conventional standard for welding and not for establishing quality in WAAM products. We took the liberty of borrowing this standard to establish a methodology to classify defective WAAM samples. ISO/ASTM 52922-F3413-19-Guide specifies the features of direct energy deposition (WAAM comes within this classification) and detailed design recommendations [22]. But ISO 5817 defines three classes for defects/porosity, where B is the most stringent defect classification (defect less than 1%), and D is classified as moderate level which is defect of less than 2.5% but greater than 1.5%, whereas C is classified as between 1 and 1.5%. This is an apt classification methodology that can be adopted here, and, hence, based on such a regime, we define three limits of defect classification. The experimental and simulated database containing information on the frequency and dimensions of the samples are used to train the algorithm. Samples can be classified into three broader categories based on the defects, and, within the algorithm, this is taken care of by implementing regression modelling; the frequencies are classed into three categories, and these correspond to the level of defects within the samples. The regression modelling takes into account several inputs including frequencies and dimensions. This implies “*Class-Good*” shall have a fixed frequency range, where each frequency of a sample corresponds to an amount of defect in them and the amount shall not exceed 2%. Beyond 2 and below 3% is classed as “*Class-Acceptable*”, which again corresponds to a frequency range. Beyond 3% is classed as “*Class-Bad*”, having its own frequency range. These frequency ranges shall be fixed based on the defect concentration, and shall be elaborated later in the Results section of this paper. The classification threshold is specified in Table 3, and this will be correlated to corresponding frequencies. To be precise, if a sample has a defect concentration of 1.5%, then a corresponding frequency shall be recorded and fed into the database.

**Figure 6:**
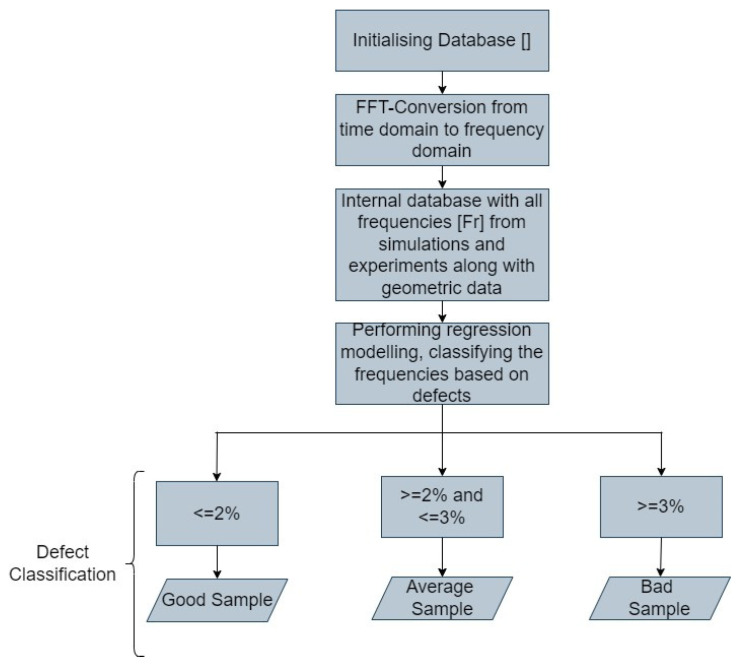
General flowchart of the algorithm implemented in this paper.

Thus, based on the explanations in the previous sub-sections, the main flowchart of the developed generalized algorithm is shown in the figure below (Figure 6). The process starts with initializing the dataset containing all time-domain signals from the equipment, also termed as raw data. Subsequently, each of these raw data is converted to frequency domain as elaborated in the previous sections and also seen in the flowchart. Next, the database is generated with all the frequencies from experiments and simulations along with the geometric data. Regression modelling is performed on the dataset as a training set, and, based on the classification threshold stated in the previous paragraph, the samples are classified based on their quality.

Lastly, the algorithm needs to be validated, and, hence, a set of testing data needs to be generated. For this purpose, an additional set of four samples were prepared and cut into the same dimensions elaborated in Section 2.1. The frequencies were collected for these samples without any porosity in them. Subsequently, they were drilled at random positions, the dimensions being the same as mentioned earlier. The frequencies were recorded along with the dimensions and the testing-set data were generated. These were then used to test the algorithm and validate its performance.

## 3. Results and Discussion

### 3.1. Density Measurements and Micrography

In comparing the two materials presented in Table 4 below, wrought and Wire Arc Additive Manufacturing (WAAM), respectively, notable differences in both the density and E-modulus are evident. The wrought material exhibits a higher density of 7990 kg/m^3^ and a correspondingly higher E-modulus of 190 GPa, indicating a denser and stiffer material compared to WAAM. On the other hand, the WAAM material has a slightly lower density of 7837 kg/m^3^ and a lower E-modulus of 169 GPa [13], suggesting a lighter and less rigid structure compared to the wrought material.

**Figure 7:**
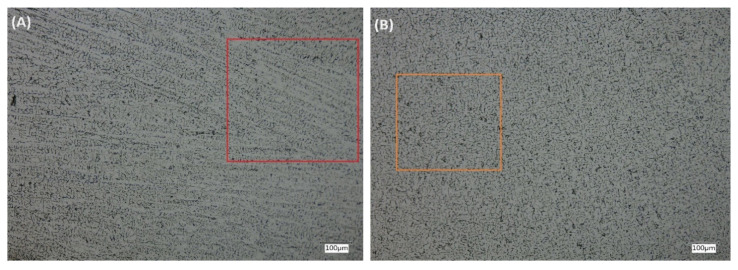

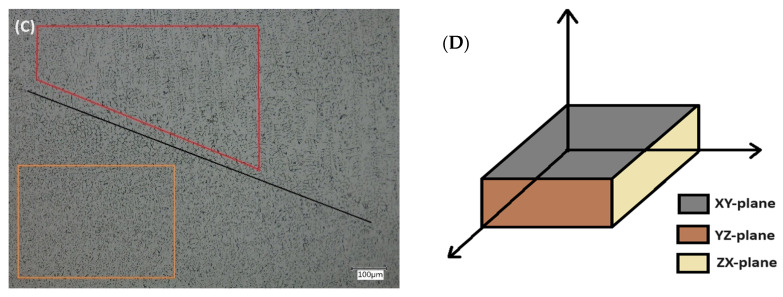
Microstructure from WAAM samples in (**A**) xy, (**B**) zx, and (**C**) yz planes, respectively; and (**D**) 3D projection of WAAM sample planes where the respective examination were conducted.

γ-austenite and δ-ferrite make up the solidification structure of a typical WAAM part, as empirically represented by the constitution diagrams by Schaeffler (1949) [23] and Delong (1974) [24]. The steel employed in this study, 316LSi, solidifies as δ-ferrite with a morphology resembling a vermicular or lath. The alignment with the direction of the heat flow and the crystallographic orientation relationship with austenite are necessary for the morphogenesis [25,26]. The morphology of δ-ferrite varies throughout the section; therefore, it displays vermicular and lathy morphologies in the fusion zone (shown as a black line in Figure 7C). A vermicular structure can be seen beneath the fusion zone in Figure 7C (highlighted in orange). Looking at this structure in the zx direction, Figure 7B shows it more clearly. Again, above the fusion zone in Figure 7C is a mixture of a columnar and globular structure (highlighted in red). When seen in the xy plane, Figure 7A makes this more evident by showcasing the columnar morphology. Consequently, there are several morphologies present in a WAAM sample, and the welding community has studied this. According to Suutala et al. (1979) [27], the composition of the weld metal affects the δ-ferrite shape. Consequently, distinct solidification modes and, subsequently, diverse morphologies are produced by differences in the material composition. A comparable morphological structure with a distinct change from vermicular to columnar structures was noted by Belotto LP et al. [25]. The microstructure being checked for this research is highly significant. The effect of a non-equilibrium microstructure affects the mechanical property of the material to a large extent, and its correlation with sound propagations needs to be studied further. For instance, the yield strength was seen to be significantly affected by the presence of a non-equilibrium micro-structure [28]. If this is true, the E-modulus is also different within the structure along the build and scanning directions, which can be ascertained from performing tensile tests. The E-modulus measured via acoustic methods should be revised for WAAM samples owing to their unique microstructure.

### 3.2. FEM-Simulated Sample Frequencies with/without Defects

**Figure 8:**
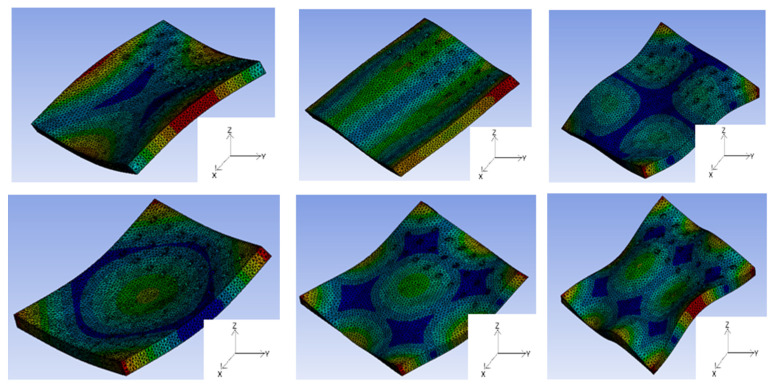

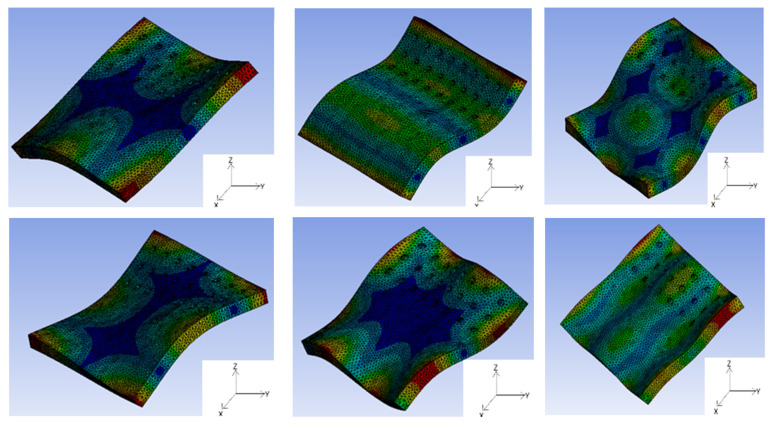
FEM-calculated modes of sample with 1.02% porosity.

Thus, based on the methodology elaborated in Section 2.3, the modal frequencies of a part with 1.02% porosity are illustrated in Figure 8. There are 12 modal frequencies shown in the figure with a maximum frequency of 36 kHz. With the mode shapes, in addition to the usual bending or flexural modes, one can observe the pumping or thickness-stretching mode, where deflections are predominant and are symmetric with respect to the mid-plane. The in-plane or extensional modes (one longitudinal displacement prevails) is also seen along with bi-in-plane modes (two longitudinal displacement acts simultaneously) [29]. The modes are a combination of Mode-1, Mode-2, Mode-3, etc., according to an ascending order usually seen in a geometrically mean part [10]. The modal frequencies vary in a mode-specific way as a function of the mechanical part structures.

Table 5 has an estimation of the sample modal frequencies based on the presence of defects. The table elaborates on the first 12 modes, where columns 2 to 12 contain the respective frequencies. From the table, the following can be briefly explained:

(a) Without defect: This information is elaborated in column 2 [0% porosity]. A parametric study was performed to ascertain the frequency shift by keeping the geometry fixed (dimensions are same as that used in experiments), while the E-modulus is varied between a range of 180–200 GPa. Finally, at 190 GPa, the frequency difference between FEM and experimental values (7030 and 7018 Hz, respectively) were narrowed down to just 0.17%. This inference serves an important function, which is identifying the correct resonant frequency from experiments and cancelling out the others.
(b) Frequency shifts with increasing defect percent: Samples with varying amounts of porosity are shown in column 3. The frequency shifts with respect to each mode can be seen as well.

**Figure 9:**
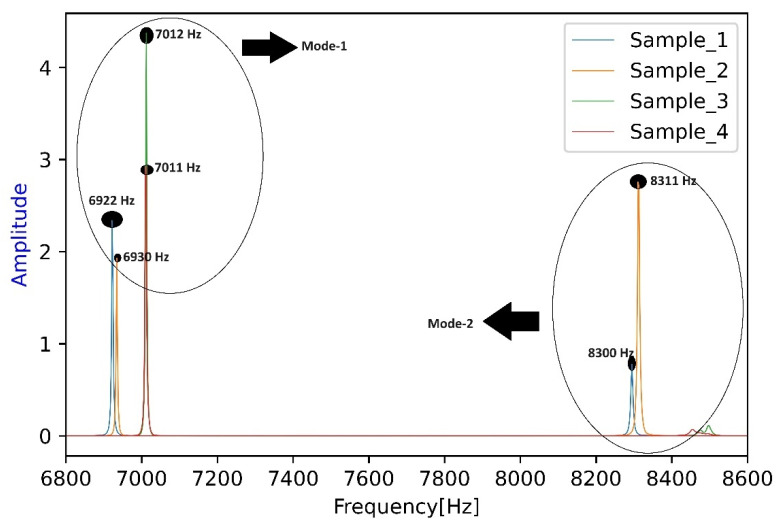
Spectrum highlighting the activation of an extra mode with the presence of other defects in an altered position.

From Table 5, Mode-1 was adopted for this study as this frequency was seen to dominate the frequency spectrum when experiments were performed, while Mode-2 was seen occasionally but with a reduced intensity. The basis for this assertion is shown pictorially below in Figure 9. Here, the frequency spectrum obtained from a rejected set of samples can be seen. The samples had the same dimensions considered in this paper, but the defect inclusion was wrongly carried out; i.e., the experimental location and dimensions of defects could not be correctly reproduced while the simulations were performed—hence, the sample were not used for further analysis. But an important observation can be seen here, where Mode-1 and Mode-2 are activated when the hole positions changed (several other reasons can also be attributed to this, along with microstructural changes). While taking a closer look into this picture, Sample-1 (marked as blue) has two active modes with 6922 Hz and 8300 Hz, respectively, where the former is dominant. Within the spectrum, only Sample-2 (marked as orange) had a dominant Mode-2, but the dominance of Mode-1 is clear as well. All the other samples had dominant Mode-1 frequencies, while Mode-2 was seen to be negligible in the case of Sample-3 and 4, respectively. Therefore, Mode-1 did not shift while the defect position changed, but rather activated other modes. From the results of these four samples, special care was taken to mark the position precisely while the defect introduction was carried out. Two important conclusions were drawn from these results: firstly, simulations help us to identify the frequencies that one intends to identify in a spectrum; and, secondly, the dominant presence of Mode-1 vis-à-vis Mode-2 was evident and, hence, adopted for this study. It should be further clarified that any of these modes can be adopted for defect detection, but special care must be taken to identify them correctly using simulations.

### 3.3. Defect Classification Based on Generated (Training-Set) Data

Time-domain signals acquired from the equipment seen in Figure 4 were transformed into their respective frequency domains for samples with varying amounts of defect concentrations. Therefore, 12 such frequencies were obtained, each corresponding to a percentage of porosity, and these are shown in Table 6. As stated earlier, if the total porosity exceeds 2.5%, then quality is considered bad as per ISO 5817:2014 (see Section 2). Thus, based on an ascending order of percentage porosity, the frequencies were checked and are elaborated in Table 6. To further reinforce the accuracy of the frequencies elaborated in Table 6, simulated frequencies of Mode-1 from Table 5 were inserted as column 5 for reference. The difference between the two hovered at 0.2%; such a difference was seen by other authors [10] when comparing the FEA data with experimental values. After finding a congruence between the experimental and FEM results, the sample quality was classified into three categories. The classification methodology is explained in Section 2.4.4 where the samples are classified for their quality based on the porosity threshold. From the table below, a porosity content up to 2% was classed as ***Good***, between 2 and 3% as ***Acceptable***, and above 3% as ***Bad***.

Table 6 is shown in the form of a figure below, where the right side of the spectrum has samples with less porosity, and, moving to the left, the porosity increases, ending with red, marked as bad (different colored peaks in the spectrum corresponds to sample frequency, and to avoid confusions only few peak frequencies are highlighted). A closer look into the spectrum reveals that the difference in the frequency shifts in the green region was approximately 2–8 Hz. While moving from right to left, there was an observable larger difference between the last two peaks within this region, i.e., a 30 Hz difference. This could be attributed to an unintended weld defect/porosity in addition to the intentionally induced porosity in the sample and these could be better understood with a CT-Scan. Further moving to left, there is one peak within the acceptable region, which was 21 Hz less than the last peak in the green region. The last peak to the left which lies in the red region had a shift of 105 Hz from the first peak (in the extreme right).

**Figure 10:**
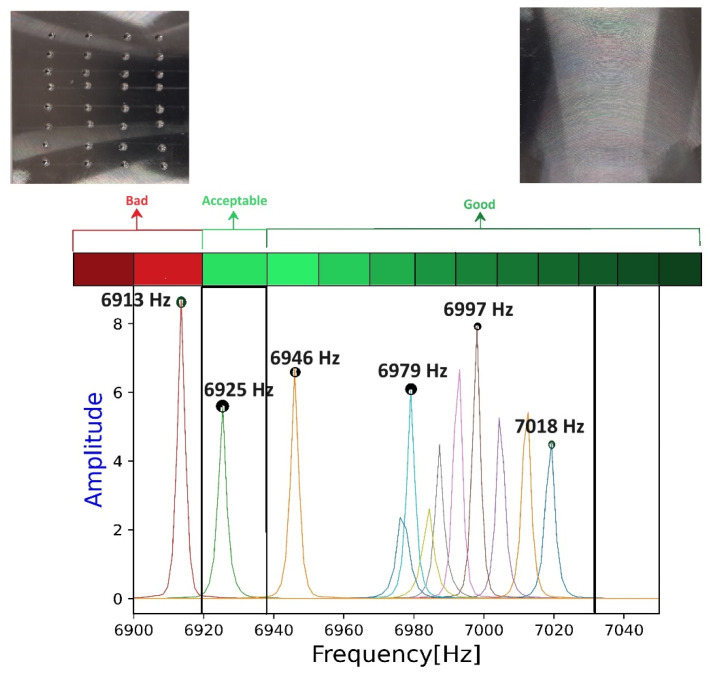
Defect classification drawn from ISO 5817.

Therefore, from Table 6 and Figure 10, the classification criteria based on frequencies can be stated. It must be noted that, from the flowchart shown in Figure 6, only defect thresholds were established but not the frequencies. Thus, frequencies seen above 6940 Hz are classified as *Good*, between 6939 Hz and 6915 Hz are classified as *Acceptable*, and below 6915 Hz are classified as *bad*. This classification is based on the thresholds defined in the standard referred to in this paper. Thus, through this method, the training-set data have been used to train the algorithm so that it can predict the outcome. Further, to test the algorithm, a testing dataset (termed as the validation set here) needs to be fed into the algorithm. This procedure is elaborated in the next section of this paper.

### 3.4. Defect Classification Based on Testing Dataset (Validation)

The testing-set data are obtained through experiments, and, for this purpose, four samples were considered. Porosities in the form of drilled holes of 1 mm diameter and 1 mm depth were introduced again. While holes were introduced at defined positions within the sample for generating the training-set data, the same methodology was not adopted while generating the testing-set data. This was because the testing-set data should be more realistic to test the reliability of the algorithm, thereby making it more versatile. Therefore, initially, the samples were subjected to RFM and their frequencies without any defects ascertained. This is shown under the leftmost column of Table 7, where the frequencies are 7016, 7014, 7017, and 7013 Hz, respectively. The same colour gradient seen in Figure 10 was adopted in Table 7.

**Figure 11:**
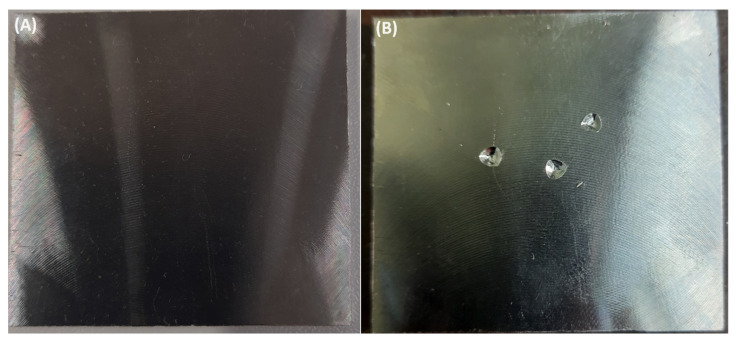
Sample-1 with and without intentional defects. (**A**) Sample before introduction of defect, (**B**) Sample with 0.2% defect concentration.

Subsequently, Sample-1 was introduced with defects as shown in Figure 11, initially with three holes, as can be seen in the figure; these are not at clearly defined locations but at random positions (but the dimensions of these were kept constant throughout this paper). This was quantified to be 0.2% porosity, and, for this porosity, the frequency seen in Table 6 was 7012 Hz. The algorithm classified it to be under the column *Good* in Table 7. Now, the porosity content in Sample-1 was increased to 4% from 0.2%, and this was classified as *Bad* in Table 6, and the same is true when seen in Table 7. Next, in Sample-2, a 2% porosity was introduced, which is a notch above *Acceptable* in Table 6. But the algorithm classified it to be *Acceptable* instead of *Good*; this incorrectness in classification can be rectified by further enhancing the training-set data through simulated results. A 0.9% porosity was introduced in Sample-3, and this was rightly classified as *Good*, and, when the porosity was increased to 4%, it was classified as *Bad*. Sample-4 too was correctly classified as *Good* and later changed to *Acceptable* when the porosity content was increased to 2% from 0.2%, respectively.

### 3.5. Future Work

In the current work, the porosities were introduced in the form of drilled holes of fixed dimensions. The dimensions of the samples along with the frequencies were used as the training and testing dataset for the algorithm. But, to make this methodology robust and the algorithm versatile, a high-end CT-Scan shall be used to quantify the porosities in the samples for different weld parameters. And the data shall be used to train the algorithm which could classify the samples based on the actual percentage porosity instead of classifying them as *Good*, *Acceptable*, and *Bad.* Later, a parametric study on frequency deviations as a function of E-modulus shall be performed; this shall be studied experimentally to ascertain the influence of anisotropy in the samples.

## 4. Conclusions

The study is an attempt to characterize flawed WAAM samples with the help of an algorithm being trained with a dataset (training set) generated from FEA and experimental methods. WAAM samples were fabricated and cut down, the latter milled-to-precision dimensioned samples. A microscopic analysis along the -xy, -yz, and -zx planes were carried out and the typical vermicular structure was seen beneath the fusion zone, and above this zone is a mixture of a columnar and globular structure. A sample without any defect in it was subjected to a resonant frequency test (RFM) to identify its resonant frequency. Later, FEM on a similar-sized sample was performed, wherein a parametric study was conducted to align the frequency with its experimental value. The modal frequencies were obtained and these were compared with experimental frequencies seen in the spectrum. Here, Mode-1 was seen to dominate to a larger extent, whereas Mode-2 was seen to a lesser extent. Mode-1 was adopted for comparison in this paper as it was observed that other modes were either lower in intensity or completely absent from the spectrum. The presence of defects was seen not to affect the first mode but rather activate the second mode. Subsequently, intentional defects were introduced into the experimental samples, based on the limits mentioned in ISO 5817 (this was performed to mimic the porosity content). These were subjected to RFM to further augment the training set with frequencies along with sample dimensions corresponding to the amount of defect in the sample. The database was used to train an algorithm (built on regression modelling), which would then sort samples based on the amount of defect in them. The detectability of the algorithm was tested with samples having a known amount of defect in them, but the locations of these defects were random. Sample-1, initially with a defect content of approximately 0.2%, was classed as *Good*, and, when the defect content was increased to 4%, the algorithm duly classified it as *Bad*. An anomaly was seen in the classification of Sample-2, where a 2% defect was introduced, which was classified as *Acceptable* by the algorithm. But a 2% defect was well within the criterion classed as *Good*. Therefore, the training set needs to be further enhanced by a simulated dataset so that the algorithm can better predict the outcome. The other two samples were classed to the relevant section based on the established criterion.

## Figures and Tables

**Table 1:** Chemical composition of wire (wt.%).

	Cr	Ni	Mo	Mn	Si	C	N	Nb	P	Fe
Wire 316LSi	18.65	11.64	2.29	1.76	0.65	0.026	0.035	0.016	0.003	64.62

**Table 2:** Material properties of the samples considered [13].

Material	Density [kg/m^3^]	E-Modulus [GPa]	Poisson’s Ratio
316LSi	7837	190	0.27

**Table 3:** Defect/porosity threshold for defect classification.

Defect/Porosity Concentratin [%]	Sample Classification
≤2	Good
≥2 and ≤3	Acceptable
≥3	Bad

**Table 4:** Density and E-modulus comparison between a conventional wrought material (316L) and WAAM (316LSi).

Sample	Density (kg/m^3^)	E-Modulus (GPa)
Wrought	7990	190
WAAM	7837	169

**Table 5:** Frequency [Hz] shift with increasing defect (Def) in [%].

	Def [%]	0%	0.22%	0.31%	0.43%	0.52%	0.65%	0.76%	0.91%	1.02%	2.04%	3.06%
Mode												
1		7030	7024	7016	7009	7007	7002	6999	6994	6991	6966	6954
2		8293	8297	8297	8296	8294	8289	8288	8289,	8287	8268	8216
3		11,644	11,647	11,642	11,635	11,628	11,619	11,612	11,608	11,602	11,565	11,517
4		11,943	11,946	11,943	11,935	11,928	11,915	11,907	11,904	11,895	11,850	11,802
5		19,610	19,612	19,597	19,579	19,570	19,571	19,553	19,538	19,528	19,461	19,383
6		20,869	20,865	20,846	20,833	20,822	20,828	20,814	20,796	20,785	20,679	20,604
7		20,950	20,953	20,951	20,948	20,946	20,943	20,942	20,940	20,937	20,797	20,663
8		22,684	22,685	22,672	22,653	22,640	22,625	22,606	22,594	22,580	22,510	22,446
9		25,130	25,133	25,118	25,102	25,090	25,089	25,072	25,058	25,045	24,927	24,827
10		33,007	32,999	32,980	32,972	32,958	32,936	32,928	32,913	32,900	32,756	32,640
11		33,576	33,571	33,538	33,512	33,493	33,504	33,479	33,444	33,424	33,299	33,218
12		36,455	36,454	36,420	36,414	36,400	36,377	36,369	36,333	36,320	36,173	36,041

**Table 6:** Sample porosity percentage correlation with frequency.

	Pores % w.r.t Surface Area	Mass [g]	Frequency-Exp [Hz]	Frequency-FEM [Hz]	Category
0	0	80.05	7018	7030	I-Good
1	0.22	80.03	7012	7024	
2	0.31	80.01	7004	7016	
3	0.43	79.99	6997	7009	
4	0.52	79.97	6992	7077	
5	0.65	79.95	6987	7002	
6	0.76	79.93	6984	6999	
7	0.91	79.92	6979	6994	
8	1.02	79.90	6976	6991	
9	2.04	79.76	6946	6966	
10	3.06	79.62	6925	6945	II-Acceptable
11	4.02	79.48	6913	6930	III-Bad

**Table 7:** Random samples having intentional defect, and the results from algorithm.

Sample No.	Classification				
	Good	Good	Good	Acceptable	Bad
1	7014	7006			6901
2	7016			6915	
3	7017		6980		6897
4	7013	7009		6933	

Note: Background colour in the table reflects the gradation used from left to right in Figure 10. The colour gradation reflects the transition of samples from Good to Bad.

## Data Availability

The data will be available upon request.

## Abbreviations

AM	Additive manufacturing
WAAM	Wire arc additive manufacturing
RFM	Resonant frequency method
ISO	International Organisation for Standardisation
FEM/FEA	Finite element method/Finite element analysis
GMAW	Gas metal arc welding
GTAW	Gas tungsten arc welding
NDT	Non-destructive testing
XCT	X-ray computer tomography
RUS	Resonant ultrasound spectroscopy
ART	Acoustic resonance testing
SCNN	Spectral convolution neural network
TS	Travel speed
WFS	Wire weed speed
CAD	Computer-aided design
FFT	Fast Fourier transform
